# Reduced monocyte and macrophage *TNFSF15*/TL1A expression is associated with susceptibility to inflammatory bowel disease

**DOI:** 10.1371/journal.pgen.1007458

**Published:** 2018-09-10

**Authors:** Arianne C. Richard, James E. Peters, Natalia Savinykh, James C. Lee, Eric T. Hawley, Françoise Meylan, Richard M. Siegel, Paul A. Lyons, Kenneth G. C. Smith

**Affiliations:** 1 Department of Medicine, University of Cambridge School of Clinical Medicine, University of Cambridge, Cambridge, United Kingdom; 2 Immunoregulation Section, Autoimmunity Branch, National Institute of Arthritis and Musculoskeletal and Skin Diseases, National Institutes of Health, Bethesda, MD, United States of America; 3 Cambridge Institute for Medical Research, University of Cambridge, Cambridge, United Kingdom; 4 Cardiovascular Epidemiology Unit, Department of Public Health and Primary Care, University of Cambridge, Cambridge, United Kingdom; 5 NIHR Cambridge BRC Cell Phenotyping Hub, Department of Medicine, University of Cambridge, Cambridge, United Kingdom; University College London, UNITED KINGDOM

## Abstract

Chronic inflammation in inflammatory bowel disease (IBD) results from a breakdown of intestinal immune homeostasis and compromise of the intestinal barrier. Genome-wide association studies have identified over 200 genetic loci associated with risk for IBD, but the functional mechanisms of most of these genetic variants remain unknown. Polymorphisms at the *TNFSF15* locus, which encodes the TNF superfamily cytokine commonly known as TL1A, are associated with susceptibility to IBD in multiple ethnic groups. In a wide variety of murine models of inflammation including models of IBD, TNFSF15 promotes immunopathology by signaling through its receptor DR3. Such evidence has led to the hypothesis that expression of this lymphocyte costimulatory cytokine increases risk for IBD. In contrast, here we show that the IBD-risk haplotype at *TNFSF15* is associated with decreased expression of the gene by peripheral blood monocytes in both healthy volunteers and IBD patients. This association persists under various stimulation conditions at both the RNA and protein levels and is maintained after macrophage differentiation. Utilizing a “recall-by-genotype” bioresource for allele-specific expression measurements in a functional fine-mapping assay, we localize the polymorphism controlling *TNFSF15* expression to the regulatory region upstream of the gene. Through a T cell costimulation assay, we demonstrate that genetically regulated TNFSF15 has functional relevance. These findings indicate that genetically enhanced expression of *TNFSF15* in specific cell types may confer protection against the development of IBD.

## Author summary

Crohn’s disease and ulcerative colitis, characterized by gut inflammation, are the two main subtypes of Inflammatory bowel disease (IBD). Over two hundred genetic loci have been identified that contribute to risk for IBD. However, functional studies are required to determine the mechanisms by which these genetic changes might affect disease risk. To date, only a few IBD loci have been functionally characterized. We focused on the IBD risk locus at *TNFSF15*, encoding the cytokine also known as TL1A. Previous work has shown that TNFSF15 enhances lymphocyte activation and promotes inflammation in animal models of disease. However, we here call into question the assumption that TNFSF15 is always a pro-inflammatory molecule by demonstrating that the IBD risk allele at *TNFSF15* is associated with decreased production of TNFSF15 by monocytes and macrophages at rest and after stimulation. In addition, we narrow the list of potential variants at *TNFSF15* responsible for controlling its expression, and we demonstrate that genetically controlled TNFSF15 can have functional impact on responding T cells. These findings both demonstrate a mechanism by which this IBD risk locus might drive disease predisposition and suggest a new protective role for TNFSF15 in maintaining the intestinal barrier.

## Introduction

Maintaining immune homeostasis in the microbiome-rich environment of the intestine is a complex process, mediated by numerous mechanisms including the physical epithelial barrier, mucin secretion, antimicrobial peptides, anti-inflammatory cytokines, regulatory cells, and IgA responses [1, 2]. Inflammatory bowel disease (IBD, including Crohn’s disease, CD, and ulcerative colitis, UC) results from a breakdown in mucosal immune homeostasis [3], but the precise mechanisms by which barrier dysfunction begins remain largely unknown. To date, genome-wide association studies (GWASs) have identified approximately 200 distinct susceptibility loci for IBD, the majority of which are associated with both CD and UC [4, 5]. A fine-mapping study used Bayesian statistical methodology to find the most probable causal variants underlying the association signal at each locus [6]. However, to fully realize the benefit of GWAS discoveries, functional studies are required to move beyond statistical associations with genetic loci and uncover the biological mechanisms behind genetic predisposition to disease. Functional studies of IBD-associated genetic variants have been performed for several loci, demonstrating that risk variants can lead to a breakdown in the intestinal barrier through both reducing (e.g *NOD2* [7–10] and *ATG16L1* [11–13]) and enhancing (e.g. *IL23R* [14–17]) gene function. Expression quantitative trait locus (eQTL) studies have found genes whose expression may be increased or decreased by IBD risk-associated variants and have highlighted the impact of cell type on the effects of a genetic variant [18–20]. In a complementary approach, investigation of gene expression patterns in monocytes and monocyte-derived macrophages revealed enrichment for genes near IBD loci among those upregulated during macrophage differentiation or stimulation [21], suggesting the importance of this cellular lineage in IBD. Investigating how IBD-associated variants influence disease susceptibility should provide insight into disease etiology and enable design of improved therapeutic or preventive strategies.

The genomic locus encoding the tumor necrosis factor superfamily member *TNFSF15* (also known as TL1A) is associated with both CD and UC in populations of multiple ethnic backgrounds [4, 5, 22, 23]. TNFSF15 is produced by a variety of tissues, the most studied of which include myeloid lineage cells, activated T cells, and endothelial cells [24]. At the cellular level, TNFSF15 costimulates both T cells and innate lymphoid cells (ILC) and promotes differentiation of IL-9-producing T cells [25–31]. Increased TNFSF15 expression has been observed systemically and at the site of inflammation in IBD and is particularly associated with active disease [32–37]. Such observations have led to the hypothesis that the allele associated with risk for IBD at *TNFSF15* might increase its expression and thereby promote inflammation. Several studies have found associations between genetic variants tagging the IBD-associated locus at *TNFSF15* and *TNFSF15* mRNA and protein expression [18, 19, 38–47]. However, the population studied, the disease status of the subjects, the cell type considered and the observed direction of effect differ between studies, leaving the mechanism by which this genetic locus confers susceptibility to IBD unclear.

To shed light on the functional consequences of the IBD susceptibility locus at *TNFSF15* we examined the association of single nucleotide polymorphism (SNP) genotype with mRNA expression in specific immune cell types in multiple cohorts of healthy individuals and patients with inflammatory diseases. We found that the IBD-associated locus is an eQTL for monocyte *TNFSF15*. The genetic signals underlying the associations with *TNFSF15* expression and IBD colocalize, suggesting that disease risk is mediated though regulation of gene expression. Importantly, we show that the IBD-protective allele at *TNFSF15* is strongly associated with *increased* monocyte *TNFSF15* mRNA in both healthy individuals and patients with inflammatory diseases. To further investigate the mechanism of this protective haplotype, we used a “recall-by-genotype” bioresource of healthy individuals from the United Kingdom. This analysis demonstrated that the IBD-protective allele is associated with increased *TNFSF15* mRNA and protein expression under a variety of stimulation contexts, as well as monocyte costimulatory capacity in acute lymphocyte activation. Through allele-specific expression measurements in individuals with breaks in the associated haplotype block, we functionally fine-mapped the expression-associated locus to upstream of the gene. Importantly, we found that association of the protective allele with increased *TNFSF15* expression was maintained in monocyte-derived macrophages, which play an important role in mucosal immunology. Thus, our findings suggest that genetically elevated TNFSF15 from monocytes or monocyte-derived cells may protect healthy individuals from the development of IBD.

## Results

### The IBD protective genotype at *TNFSF15* is associated with increased *TNFSF15* expression in circulating monocytes

**Fig 1:**
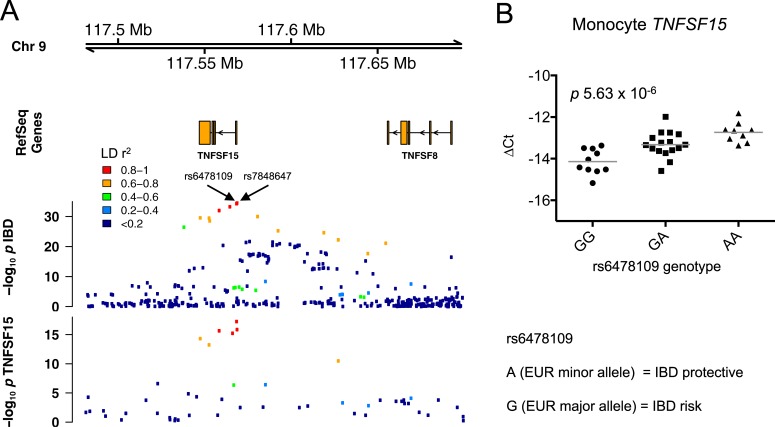
The IBD-protective rs6478109:A allele is associated with increased *TNFSF15* expression. (**A**) Regional association plots for IBD (European ancestry cohort from Liu *et al* [4]) and *TNFSF15* expression (n = 39 healthy individuals and 80 IBD patients). (**B**) *TNFSF15* mRNA expression was measured in *ex vivo* monocytes relative to *B2M* by qPCR in a separate cohort of healthy individuals and plotted versus rs6478109 genotype (GG, n = 10; GA, n = 16; AA, n = 9). Expression is reported as ΔCt, each point represents an individual, lines represent mean values, and *p* value is from linear regression.

We previously identified an association between genotype at the *TNFSF15* IBD susceptibility locus and *TNFSF15* mRNA expression in peripheral blood monocytes in both healthy individuals and newly diagnosed IBD patients from the UK [18, 19] (Fig 1A, S1 Fig). We also confirmed this association in a cohort of British patients with another immune-mediated disease, anti-neutrophil cytoplasmic antibody (ANCA)-associated vasculitis [18] (S1 Fig). The SNP most associated with *TNFSF15* expression was rs6478109 [19], which is located 358 base pairs upstream of *TNFSF15* and has a minor allele frequency (MAF) of 32.5% in individuals of European descent. An IBD GWAS trans-ancestry analysis by Liu *et al* indicated that the SNP most strongly associated with IBD in individuals of European descent at this locus is rs7848647 [4], which is 280 base pairs upstream of rs6478109 and in near complete linkage disequilibrium (LD) with it (r^2^ = 0.995 in 1000 Genomes Phase 3 European cohort). Comparison of the regional association plots for the *TNFSF15* eQTL and IBD susceptibility revealed similar patterns of association (Fig 1A). In order to formally evaluate whether the eQTL and the disease association signal are driven by the same causal variant or two distinct variants in LD, we performed colocalization testing [48]. Our results indicated that the colocalization was highly likely (posterior probability of a shared underlying causal variant was 0.998).

rs6478109 genotype is also associated with *TNFSF8* expression in whole blood [49] and monocytes [50], and additional monocyte *TNFSF8* eQTLs have been found in this genomic region [18–20, 51]. However, rs6478109 is not the SNP most associated with *TNFSF8* expression in any of these studies. Comparison of the patterns of genetic association across this locus with IBD and *TNFSF8* expression in our previous monocyte eQTL data [19] demonstrated stark differences (S2 Fig), suggesting that *TNFSF8* is unlikely to be the causal gene at this locus. Colocalization testing confirmed that the IBD association and *TNFSF8* eQTL signals were very unlikely to be driven by a shared causal variant (posterior probability 0.092).

These analyses indicate that IBD susceptibility may be mediated by changes in *TNFSF15* expression. We therefore performed further functional examination of the locus using a recall-by-genotype study design in an independent bioresource of healthy individuals recruited from the region around Cambridge, UK. We confirmed the association of rs6478109 with *TNFSF15* mRNA in peripheral blood monocytes (*p* 5.63 x 10^−6^, Fig 1B, S1 Table). Importantly, in both cohorts of healthy volunteers and the cohorts of IBD and ANCA-associated vasculitis patients, the IBD protective allele (A, the minor allele in the European population) was consistently associated with increased expression of *TNFSF15* (Fig 1, S1 Fig). In contrast to previous speculation that TNFSF15 predisposes to inflammatory disease, this suggests a novel protective role for this cytokine in preventing the development of human IBD.

**Fig 2:**
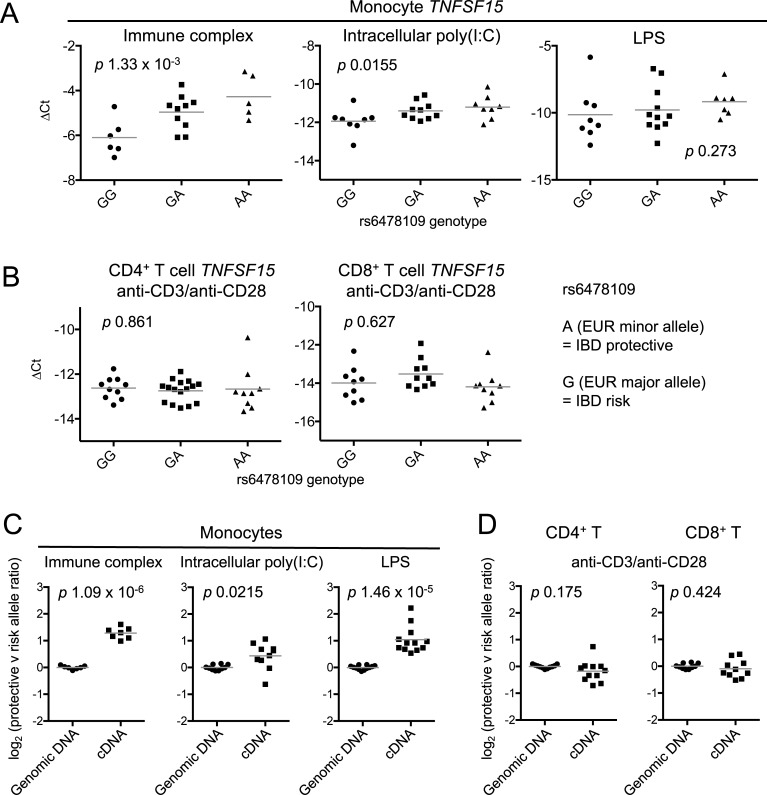
Monocyte but not T cell *TNFSF15* expression is associated with genotype after stimulation. (**A**) *TNFSF15* mRNA expression was measured relative to *B2M* in monocytes stimulated for 4 hours with immune complex (rs6478109 genotype: GG, n = 6; GA, n = 10; AA, n = 5), intracellular poly(I:C) (GG, n = 8; GA, n = 10; AA, n = 8), or LPS (GG, n = 8; GA, n = 11; AA, n = 7). Expression is reported as ΔCt, each point represents an individual, lines represent mean values, and *p* values are from linear regression. (**B**) As (A) for CD4^+^ (GG, n = 10; GA, n = 16; AA, n = 9) and CD8^+^ (GG, n = 9; GA, n = 10; AA, n = 9) T cells stimulated for 24 hours. (**C**) Allelic ratios in cDNA from stimulated cells from heterozygous individuals are compared with those in genomic DNA from the same individuals: monocytes stimulated with immune complex (n = 7), intracellular poly(I:C) (n = 10) and LPS (n = 12), and (**D**) anti-CD3/anti-CD28-stimulated CD4^+^ (n = 11) and CD8^+^ (n = 10, genomic DNA as for panel (C) intracellular poly(I:C)) T cells. Ratios for immune complex- and LPS-stimulated monocytes and stimulated CD4^+^ T cells were measured using tag SNP rs4246905 (ratio of T/C reported), while ratios for poly(I:C)-stimulated monocytes and stimulated CD8^+^ T cells were measured using tag SNP rs4263839 (ratio of A/G reported). All individuals were heterozygous at both rs6478109 and the tag SNP. rs4263839:A and rs4246905:T are in phase with the IBD-protective allele rs6478109:A in 1000 Genomes EUR subjects. Lines represent mean values and *p* values are from Welch’s t-test.

*TNFSF15* expression is rapidly upregulated in myeloid cells by stimulation via pattern recognition receptors, such as toll-like receptor ligands, and Fc receptors (FcRs) [29, 52, 53]. Although resting T cells minimally express *TNFSF15*, T cell receptor (TCR) stimulation results in robust expression [29, 54]. To examine the effect of cellular stimulation on the association of genotype with *TNFSF15* expression, we stimulated monocytes and T cells for 4 and 24 hours, respectively (time courses depicted in S3 Fig). In monocytes stimulated with immune complex and intracellular poly(I:C), the protective allele was associated with higher levels of *TNFSF15* mRNA (*p* 1.33 x 10^−3^ and *p* 0.0155, respectively), similar to results in unstimulated cells, while LPS-stimulated monocytes showed a comparable trend but with more variability (*p* 0.273, Fig 2A). A formal comparison of the eQTL in unstimulated versus stimulated monocytes revealed no significant change in the magnitude of the effect of genotype on gene expression following stimulation (Methods, S4 Fig, S1 Table). In T cells stimulated via the TCR with anti-CD3 and anti-CD28, there was no association between rs6478109 genotype and *TNFSF15* expression (CD4^+^ T cells *p* 0.861, CD8^+^ T cells *p* 0.627, Fig 2B). Thus rs6478109 is a monocyte eQTL in which the IBD risk allele is associated with reduced *TNFSF15* expression both at baseline and after stimulation.

Inter-individual differences apart from SNP genotype at the *TNFSF15* locus might influence gene expression and thereby obscure genotype-dependent differences. Heterozygotes present a unique opportunity to control for this variability as the ratio of allelic expression can be measured within each individual using allele-specific expression (ASE) assays. rs6478109 is in LD with several intronic SNPs measurable in amplified pre-mRNA, two of which we used to examine ASE (S5 Fig). Due to low *TNFSF15* pre-mRNA expression in unstimulated cells, allelic ratio measurements were only feasible in stimulated cells. In accordance with the allelic dosage effect observed in Fig 2A, such that *TNFSF15* expression increased with more copies of the IBD-protective rs6478109:A allele, heterozygous monocytes stimulated with immune complex or intracellular poly(I:C) showed significant allelic imbalance favoring the protective allele (*p* 1.09 x 10^−6^, *p* 0.0215, respectively, Fig 2C left and middle panels). Although *TNFSF15* expression in LPS-stimulated monocytes was not associated with allelic dosage by standard eQTL analysis (Fig 2A), in the internally controlled environment of heterozygous individuals, we did find significant ASE (*p* 1.46 x 10^−5^, Fig 2C right panel). In contrast, once again, stimulated T cells showed no allelic imbalance (CD4^+^ T cells *p* 0.175, CD8^+^ T cells *p* 0.424, Fig 2D and S2 Table), confirming the monocyte-specific nature of the *TNFSF15* eQTL.

**Fig 3:**
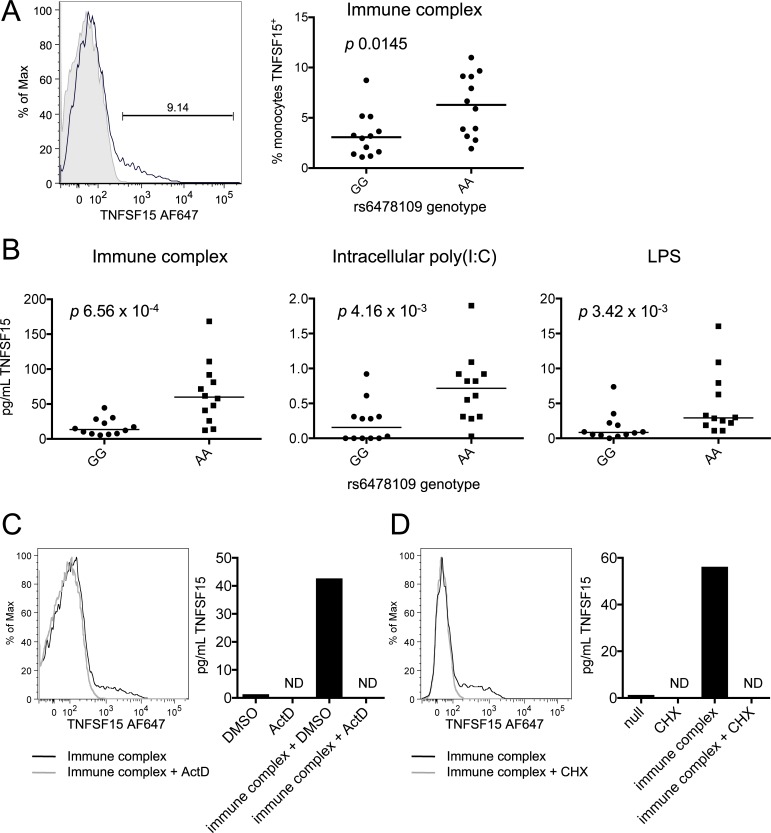
Genotype is associated with *de novo* TNFSF15 protein production in stimulated monocytes. (**A**) An example of gating for TNFSF15^+^ monocytes after 4 hours immune complex stimulation is depicted (left). Black line = monoclonal anti-TNFSF15; grey shading = isotype control. Percentages of TNFSF15^+^ monocytes after immune complex stimulation are quantified for GG (n = 12) and AA (n = 12) homozygous individuals (right). (**B**) Soluble TNFSF15 was measured in supernatants of monocytes from GG (n = 12) and AA (n = 12) homozygous individuals after the indicated 4-hour stimulations by custom anti-TNFSF15 Bio-Plex assay. *p* values are from Mann-Whitney test. (**C**) Monocytes were stimulated with immune complex for four hours in the presence of actinomycin D (ActD) or dimethyl sulfoxide (DMSO) control. ND indicates none detected. (**D**) As (C) in the presence or absence of cycloheximide (CHX). (C) and (D) are representative of two independent experiments each.

The most direct mechanism by which *TNFSF15* mRNA expression could influence IBD susceptibility would be through controlling TNFSF15 protein levels. Stimulated monocytes express transmembrane TNFSF15 protein, but it is rapidly cleaved from their surfaces. TNFSF15 expression is thus measurable both on the cell surface and in the supernatant. To determine if the *TNFSF15* eQTL extended to the protein level, we first measured surface TNFSF15 expression in immune-complex stimulated monocytes from homozygous individuals. Individuals homozygous for the IBD protective allele exhibited increased cell surface TNFSF15 (*p* 0.0145, Fig 3A). We next looked at soluble TNFSF15 in the supernatants of monocytes stimulated with immune complex, intracellular poly(I:C), or LPS. In the absence of stimulation, soluble TNFSF15 levels in monocyte supernatants were low (around the limit of detection) regardless of genotype. In contrast, under all stimulation conditions, soluble TNFSF15 was significantly increased in supernatants of cells from IBD protective allele homozygotes (immune complex *p* 6.56 x 10^−4^, intracellular poly(I:C) *p* 4.16 x 10^−3^, LPS *p* 3.42 x 10^−3^, Fig 3B). Thus, the levels of TNFSF15 both on the cell surface and in solution were associated with genotype. Of note, total serum TNFSF15 protein levels were not associated with genotype (*p* 0.962, S6A Fig), suggesting that the effect of genotype on monocyte TNFSF15 levels is relevant primarily in local cellular contexts. Secretion of other inflammatory cytokines from these monocytes was not associated with rs6478109 genotype at the same time-point, demonstrating the cis-specificity of the *TNFSF15* eQTL (S6B–S6C Fig). To confirm that secreted TNFSF15 originated from newly synthesized protein, we stimulated monocytes with immune complex in the presence of either actinomycin D to block transcription (Fig 3C) or cycloheximide to block translation (Fig 3D). Both treatments resulted in loss of detectable protein, indicating that the effects of genotype on TNFSF15 protein expression were due to differences in *de novo* transcription and translation of *TNFSF15*.

### Fine-mapping narrows the functional variant in the *TNFSF15* locus to upstream of the gene

**Fig 4:**
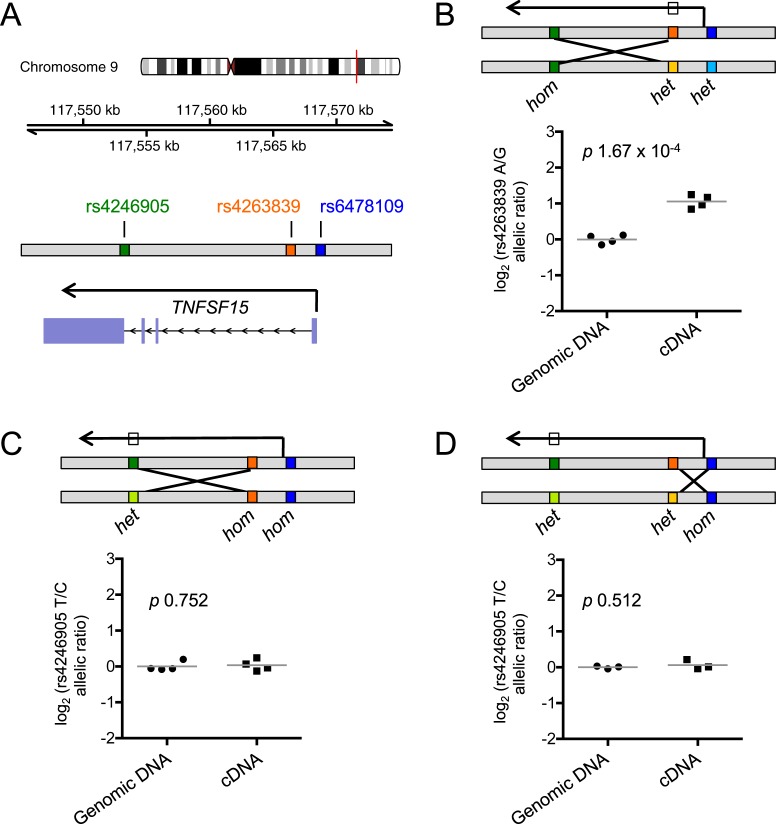
The eQTL causal SNP resides upstream of *TNFSF15*. (**A**) The *TNFSF15* locus on chromosome 9 is depicted, marking SNPs used for functional fine-mapping. (**B**) Immune complex-stimulated monocytes from individuals heterozygous at rs6478109 and rs4263839 but homozygous at rs4246905 (n = 4) were examined for allele-specific expression. The box on the transcription arrow indicates the SNP used for measuring allelic imbalance. (**C**) As (B) with individuals homozygous at rs6478109 and rs4263839 but heterozygous at rs4246905 (n = 4). (**D**) As (B) with individuals homozygous at rs6478109 but heterozygous at rs4263839 and rs4246905 (n = 3). Lines represent mean values and p values are from Welch’s t-test.

Fine-mapping of genotype-phenotype associations generally requires a large number of samples to achieve the power necessary to statistically infer probable causality for one SNP over another in high LD. In order to solve this problem with a limited number of samples, we again leveraged the power of the controlled environment within heterozygous individuals. We examined ASE in immune complex-stimulated monocytes from healthy volunteers recruited specifically for having genetic cross-over events in the *TNFSF15* haplotype block, such that they were heterozygous for certain SNPs but homozygous for others (Fig 4A). An earlier IBD meta-analysis by Jostins *et al* identified rs4246905 in the third intron of *TNFSF15* as the tag SNP for disease association at this locus in European individuals [5], whereas the more recent trans-ancestry analysis described above by Liu *et al* [4] identified the upstream SNP rs7848647 as the most associated in the same population. To narrow the location of the eQTL causal variant to either the upstream or downstream portion of the gene, we first examined ASE in individuals heterozygous at rs6478109 in the promoter and rs4263839 in the first intron but homozygous at rs4246905 in the third intron. These individuals maintained ASE of *TNFSF15* (*p* 1.67 x 10^−4^, Fig 4B), indicating that rs4246905 is not causal and suggesting that the SNP influencing *TNFSF15* expression is in greater LD with the upstream SNPs than with rs4246905. Confirming this finding, individuals heterozygous at rs4246905 but homozygous at the two upstream loci showed no ASE (*p* 0.752, Fig 4C).

Examining the LD structure of variants in the *TNFSF15* locus, we found 17 variants that are in greater LD with the upstream SNPs rs6478109 and rs4263839 than with the downstream SNP rs4246905, and that are in higher LD with these upstream SNPs than is rs4246905 (S3 Table). To further distinguish between these potential causal variants, we identified individuals homozygous at the rs6478109 promoter SNP but heterozygous at rs4263839 in the first intron and measured *TNFSF15* ASE in immune-complex-stimulated monocytes. These samples lacked ASE (*p* 0.512, Fig 4D), indicating that rs4263839 is not the causal variant. Only three variants identified in the 1000 Genomes Phase 3 European cohort in high LD with rs6478109 (r^2^ > 0.8) are in higher LD with rs6478109 than with the eliminated SNP rs4263839. These are rs6478109 itself, rs7848647 (the most significant IBD-associated variant in Europeans in Liu *et al* [4]) and rs10817678, all of which are located upstream of *TNFSF15*. Two of these, rs6478109 and rs7848647, are located close to the promoter of *TNFSF15*, in a region containing a cluster of transcription factor binding sites, active chromatin marks and enhancers, making them the leading candidates for the causal variant (S7 Fig). These two SNPs were completely linked in all individuals recruited for ASE measurement in Figs 2 and 4.

### Genetically modulated TNFSF15 expression affects strength of costimulation

To examine the phenotypic consequences of variation in *TNFSF15* expression, we performed comprehensive immunophenotyping of T cells, B cells, monocytes, dendritic cells, and NK cells from peripheral blood of individuals homozygous for the rs6478109 polymorphism. We found no association between genotype and cell population frequencies (S8 and S9 Figs).

**Fig 5:**
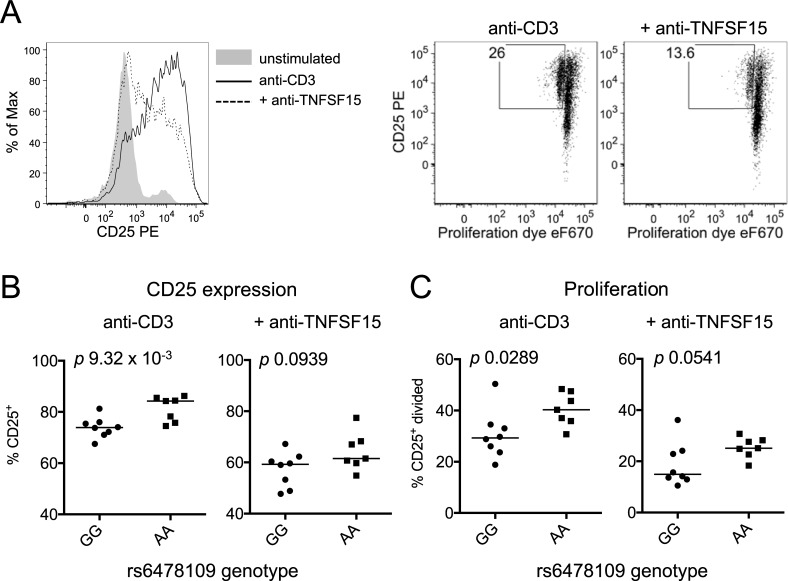
Genetically regulated TNFSF15 controls costimulatory capacity. (**A**) CD8-depleted PBMC were stained with proliferation dye eFluor670 and stimulated with 1 μg/mL anti-CD3 with and without 10 μg/mL anti-TNFSF15 for 48 hours. Flow cytometry plots depict cells gated on live, CD4^+^ cells. (**B**) CD25^+^ cells were measured as in (A) in samples from rs6478109 genotyped individuals (GG, n = 8; AA, n = 7). (**C**) CD25^+^ divided cells were measured in the same samples as in (B). *p* values from Mann-Whitney test.

To test whether genetically driven variation in *TNFSF15* expression under acute stimulation would result in differences in responding cell populations, we measured the effects of endogenous TNFSF15 on T cell activation. TNFSF15 costimulation promotes T cell proliferation and upregulation of the IL-2 receptor alpha chain (CD25), particularly under low levels of TCR stimulation [26, 29, 30]. We therefore examined CD4^+^ T cell proliferation in stimulated peripheral blood mononuclear cells from individuals homozygous for the *TNFSF15* expression-associated variant rs6478109. CD8^+^ T cells make up a highly variable proportion of peripheral blood cells across individuals and these cells respond to TCR stimulation by making and consuming IL-2, which can affect CD4^+^ T cell proliferation. To avoid this confounding factor, we depleted peripheral blood mononuclear cells of CD8^+^ T cells before culturing the remaining cells (including CD4^+^ T cells and monocytes) with low level TCR stimulation for two days. Blocking TNFSF15 signaling with an antagonistic antibody resulted in decreased CD25 expression and proliferation of CD4^+^ T cells (Fig 5A), demonstrating the impact of endogenous TNFSF15 in this setting. Samples from individuals homozygous for the IBD protective allele that is associated with increased monocyte TNFSF15 exhibited significantly increased CD25 expression and proliferation of CD4^+^ T cells compared to individuals homozygous for the IBD risk allele (*p* 9.32 x 10^−3^, *p* 0.0289, respectively, Fig 5B and 5C). The differences in CD25 expression and proliferation were reduced with addition of anti-TNFSF15 such that these activation markers were no longer statistically significantly elevated in protective allele homozygotes (CD25 expression *p* 0.0939, proliferation *p* 0.0541, Fig 5B and 5C). While this system does not recapitulate the complex intestinal environment of the pre-morbid at-risk individual, it demonstrates that genetically regulated *TNFSF15* expression can influence the ability of monocytes to costimulate responding lymphocytes, confirming the functional relevance of this *TNFSF15* eQTL.

### Monocyte-derived macrophages also exhibit allelic imbalance favoring the IBD protective allele

**Fig 6:**
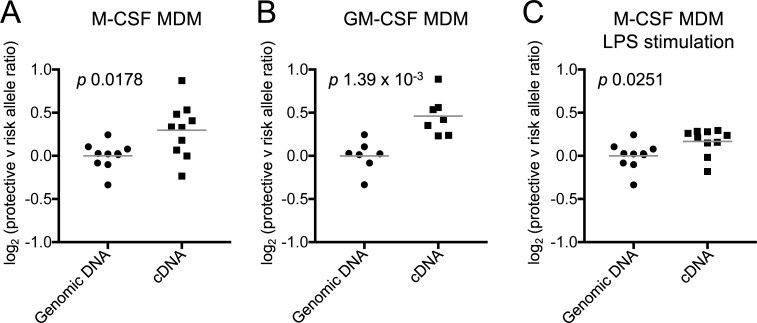
Monocyte-derived macrophages also favor expression of the IBD-protective allele. (**A**) Peripheral blood monocytes from rs6478109 heterozygotes were differentiated into macrophages in the presence of M-CSF (n = 10) or (**B**) GM-CSF (n = 7) before examining allele-specific expression. (**C**) Macrophages derived in (A) were stimulated with LPS for 4 hours before examining allele-specific expression (n = 10, genomic DNA as for panel (A)). ASE was measured at rs4263839 (ratio of A/G reported) in the first intron of *TNFSF15*. All individuals were heterozygous at both rs4263839 and rs6478109. rs4263839:A is in phase with the IBD-protective allele rs6478109:A in 1000 Genomes EUR subjects.

Intestinal CX_3_CR1^+^ mononuclear phagocytes derived from circulating monocytes [55–57] are likely to be the most relevant producers of TNFSF15 in the context of gut immune homeostasis [31]. To test whether phagocytic cells derived from peripheral blood monocytes maintain genotype-dependent *TNFSF15* expression, we differentiated monocyte-derived macrophages (MDMs) from heterozygous individuals and measured ASE. MDM differentiated in the presence of M-CSF and GM-CSF both exhibited significant allelic imbalance favoring the minor, IBD-protective allele (*p* 0.0178 and *p* 1.39 x 10^−3^, respectively, Fig 6A and 6B). ASE was also maintained after stimulation of M-CSF-derived MDM with LPS for 4 hours (*p* 0.0251, Fig 6C). To confirm these findings in a second cohort, we mined publicly available data from an MDM eQTL study by Nedelec *et al* [46]. These data demonstrated a significant *TNFSF15* eQTL at rs6478109 in resting MDM and after Salmonella infection (S10 Fig). The direction of effect was concordant with our monocyte and MDM data, such that expression of *TNFSF15* increased with more copies of the IBD-protective allele rs6478109:A. These results demonstrate that the IBD-protective allele increases *TNFSF15* expression in macrophages as well as monocytes, and is therefore likely to be relevant to the gut environment.

## Discussion

Understanding the mechanisms by which disease susceptibility variants influence disease risk is a key challenge of the post-GWAS era. Exploring the consequences of disease risk variants in healthy individuals allows examination of their effects in the pre-disease state and avoids the potential confounding effects of treatment and disease itself. Use of a “recall-by-genotype” bioresource enables investigation of specific polymorphisms with balanced experimental designs and facilitates in-depth investigation of causal quantitative trait loci by specific recruitment of individuals with breaks in common LD blocks. Here we used such a bioresource to investigate the functional consequences of an IBD-associated genetic variant and perform functional fine-mapping. We have demonstrated that the IBD-associated locus at *TNFSF15* harbors an expression-associated polymorphism in which the rs6478109:A IBD protective allele is associated with increased monocyte *TNFSF15* expression and lymphocyte costimulatory activity. Interestingly, *TNFSF15* expression in stimulated T cells was not significantly associated with SNP genotype at this locus (discussed further in S1 Text), suggesting the importance of cellular context in utilization of the particular *cis* regulatory element that this polymorphism affects. Through ASE assays in cells from individuals specifically recruited for haplotype cross-over events, we refined the location of the causal variant for gene expression to the upstream region of the gene. Colocalization testing indicated that the eQTL and IBD association are very likely to be due to the same causal variant. This suggests that the mechanism by which SNP genotype at *TNFSF15* influences IBD susceptibility is through altering *TNFSF15* expression. Our work provides an example of how functional studies not only uncover the phenotypic effects of genetic variation but can also complement statistical methods for mapping disease association.

The IBD fine-mapping study by Huang *et al* examined the *TNFSF15* locus, detailing technical difficulties with their genotyping of an indel in the region [6]. The three typed variants that interrogated the indel (chr9:117571294, chr9:117571293, and rs59418409) were assigned posterior probabilities of being causal 0.40, 0.40, and 0.11, respectively. On the basis that these three variants in fact represent a single indel (now annotated as rs35396782), the authors then summed the probabilities and concluded that this indel is the likely causal variant for IBD risk. The rs35396782 indel is 2885 base pairs upstream of *TNFSF15* and in LD (r^2^ = 0.817) with rs6478109 (S3 Table and S11 Fig). Our analysis cannot exclude the possibility that this indel is causal for the eQTL, but the LD patterns in our functional fine-mapping and the presence of active chromatin marks in the region of the promoter SNPs rs6478109 and rs7848647 favor these candidates. Robust genotyping of the indel will be necessary to draw conclusions about its association with both IBD and *TNFSF15* expression.

Previous studies describing gene expression association with genotype at *TNFSF15* have yielded conflicting results [38–45] (discussed further in S1 Text). Our data unequivocally show that the IBD risk allele is associated with decreased monocyte *TNFSF15* expression. Use of three genotyping platforms, two mRNA measurement technologies, and two protein measurement methods ensures the robustness of our results. In support of our findings, mining of the GTEx project database [45, 47] reveals a significant *TNFSF15* eQTL at rs6478109 in whole blood with the same direction of effect that we observed. Of relevance to IBD, the GTEx database also includes a nominally significant eQTL (*p* <10^−3^) with the same direction of effect in sigmoid colon.

In the gut, peripheral blood monocytes differentiate into macrophages, which are critical for maintaining mucosal immune homeostasis [58]. A recent study by Baillie *et al* posited that monocyte maladaptation to macrophage differentiation and activation in the gut environment is an important driver of IBD [21]. Examination of the supporting information for their study reveals that *TNFSF15* is strongly upregulated during this process. A key question is whether the *TNFSF15* monocyte eQTL is maintained in macrophages and is therefore likely to be relevant to IBD pathogenesis. A previous study by Hedl *et al* reported association of the *TNFSF15* risk haplotype SNP rs6478108:A (in phase with rs6478109:G, LD r^2^ 0.917, S1 Fig) with increased *TNFSF15* expression in M-CSF-differentiated MDM [41]. In contrast, we observed the opposite result in these cells (Fig 6), demonstrating ASE that was directionally concordant with our findings in monocytes. Through further ASE assays in macrophages from multiple differentiation and stimulation conditions, we demonstrated that the IBD protective allele is consistently preferentially expressed. We corroborated our findings by mining publicly-available data from a recent eQTL study in MDM with and without Salmonella infection, confirming that the IBD risk allele is associated with *lower* MDM *TNFSF15* expression [46]. We thus clearly establish that, in both monocytes and macrophages, genetic predisposition to lower *TNFSF15* expression is associated with IBD risk.

The association we have identified may at first seem counterintuitive given the known functions of TNFSF15. In IBD, TNFSF15 is generally considered an inflammatory marker, with TNFSF15 expression levels increasing with IBD activity [32–37]. However, such observational studies cannot distinguish causal effects from associations arising from confounding factors or the consequences of IBD (reverse causation). In animal models of inflammatory disease including colitis, asthma, arthritis, and experimental autoimmune encephalomyelitis, genetic or antibody-mediated disruption of TNFSF15 signaling through its cognate receptor TNFRSF25 (also known as DR3) generally leads to reduced pathology [29, 54, 59–63], but it is important to remember that these animal studies usually measure disease course and are poor models for disease susceptibility. At the cellular level, TNFSF15 generally promotes cytokines associated with inflammation, such as IL-2 and IFNγ from T cells, and IL-13 and IL-5 from ILC2 [26–28, 52, 64]. Indeed, we find that genetically-driven TNFSF15 enhances T cell activation in our *in vitro* assay. Despite this inflammatory role, studies have highlighted that TNFSF15 may be more pleiotropic than originally thought, costimulating lymphocytes that control both pro- and anti-inflammatory activities. Jia *et al* demonstrated a protective role for TNFSF15-TNFRSF25 interaction in acute DSS colitis and clearance of gut *Salmonella enterica* infection via maintenance of regulatory T cells [65], suggesting that TNFSF15 may be protective in certain contexts of intestinal inflammation. Additionally, as well as T cells and ILC2, TNFSF15 can also costimulate group 3 innate lymphocytes (ILC3), which reside in the gut and respond to TNFSF15 with enhanced IL-22 production [31, 66]. IL-22 promotes gut barrier maintenance in both infectious and non-infectious contexts [67–69]. Thus, there is also the potential for TNFSF15 to play a protective role in the gut through costimulation of ILC3.

Reduced gene expression driven by the IBD risk allele at *TNFSF15* is in line with several other IBD risk variants that reduce protein function and lead to intestinal barrier disruption. For example, the T300A variant of *ATG16L1* reduces autophagy in intestinal Paneth cells, dampening antimicrobial activity [11–13]. Likewise, while multiple mechanisms have been posited for the association of variants in the *NOD2* locus with CD [70], disease-associated coding polymorphisms were found to reduce cellular responsiveness to peptidoglycan ligands [9] and were associated with decreased expression of Paneth cell antimicrobial peptides [10] and defective anti-bacterial responses by dendritic cells [8]. The reduction in monocyte and macrophage expression of *TNFSF15* that we find associated with the IBD risk allele suggests that TNFSF15 may also promote intestinal homeostasis in the pre-morbid state.

## Materials and methods

Additional methods are included in S1 Text.

### Presentation of previous eQTL data

We present peripheral blood monocyte eQTLs for *TNFSF15* from a previous study using samples from 39 healthy individuals and 80 patients with IBD [19], as well as a study using 45 patients with anti-neutrophil cytoplasmic antibody-associated vasculitis [18]. Gene expression data was measured on the Affymetrix Human Gene 1.1 ST Array. Microarray mRNA expression was normalized by robust multiarray averaging using the oligo package [71] and adjusted with PEER [72]. IBD patient and healthy control genotypes were measured using the Illumina Human OmniExpress12v1.0 BeadChip, and vasculitis patients were genotyped using the Affymetrix SNP6.0 Array. eQTL testing was performed using a score test implemented in the GGtools Bioconductor package [73]. Genomic locus plots were generated using the Gviz Bioconductor package [74] with RefSeq annotation for genes in the hg19 genome build.

### Colocalization testing

Colocalization testing was performed using the coloc R package v2.3–1 [48]. We used the coloc.abf function and the default priors (prior probability that a SNP is associated with trait 1 = 1 x 10^−4^, prior probability that a SNP is associated with trait 2 = 1 x 10^−4^, prior probability that a SNP is associated with both traits = 1 x 10^−5^). Summary statistics for association of *TNFSF15* and *TNFSF8* expression with genotype (regression coefficients and variances) were calculated through linear regression (lm function in R) using the genotype and expression data from our previous eQTL study of healthy controls and patients with IBD [19]. For the IBD GWAS data, we used summary statistics for the European cohort from the GWAS plus Immunochip trans-ancestry MANTRA meta-analyses by Liu et al [4] (downloaded from the International Inflammatory Bowel Disease Genetics Consortium’s website, url https://www.ibdgenetics.org/, link “Latest combined GWAS and Immunochip trans-ancestry summary statistics”, file “IBD_trans_ethnic_association_summ_stats_b37.txt.gz”).

### Sample collection

Peripheral blood samples from healthy volunteers were obtained through the Cambridge BioResource. Ninety-six blood samples were taken from 90 separate volunteers recruited based on relevant genotype at rs6478109 and/or rs4246905. All recruited individuals were Caucasian, between 18 and 65 years of age. Volunteers self-declared that they were free from autoimmune disease, cancer and human immunodeficiency virus. No individuals took regular systemic immunomodulatory therapy for at least one year before recruitment. During the recruitment process, volunteer samples were grouped by genotype, and investigators were blinded as to which group corresponded to which genotype. Derived numeric data from experiments utilizing Cambridge BioResource samples are included in S1 Data.

### Whole blood processing

Whole blood was collected in CPDA tubes and passed over a Histopaque-1077 (Sigma Aldrich) gradient to separate peripheral blood mononuclear cells (PBMC). Where indicated, cell types were separated first into the CD14^+^ monocyte fraction and CD14^-^ fraction by positive selection with human CD14 MicroBeads and LS columns (Miltenyi Biotec), according to the manufacturer’s protocol. The negative fraction was then enriched for CD4^+^ T cells or CD8^+^ T cells with human CD4 or CD8 MicroBeads (Miltenyi Biotec) in the same manner. For CD8-depleted PBMC, cells were separated with CD8 MicroBeads (Miltenyi Biotec) and the negative fraction collected. Purity of separated cell subsets was examined by flow cytometry as described in the “Cell subset purity QC” section. For eQTL and ASE measurements, eighty monocyte samples were sorted for use in various assays, all with over 60% purity and a median purity of 74%; thirty-five CD4^+^ T cell samples were sorted, all with over 92% purity and a median purity of 97%; thirty-four CD8^+^ T cell samples were sorted, all with purity over 70% and a median purity of 90%; sixteen PBMC samples were depleted of CD8^+^ T cells, all demonstrating less than 7% CD8^+^ T cells remaining. One CD8-depleted PBMC sample was excluded on the basis of a CD8-intermediate CD3^+^ population composing 13% of the PBMC population after depletion.

### Cell subset purity QC

Purity of cell subsets was determined by flow cytometry. Cells were blocked with FcR Blocking Reagent (Miltenyi) and stained with anti-human CD14-PE (BD Biosciences); anti-human CD3-AmCyan, -FITC or–PE (clone SK7 or UCHT1, BD Biosciences); anti-human CD4-FITC (clone RPA-T4, BD Biosciences); and/or anti-human CD8-APC (clone RPA-T8, BD Biosciences) or -eFluor 450 (clone SK1, eBioscience). Flow cytometry was performed on a BD LSR Fortessa (BD Biosciences) and data analyzed in FlowJo (FlowJo, LLC).

### *In vitro* cell stimulations

All stimulations took place in complete medium, composed of RPMI with 10% FCS, 10 mM HEPES, 1x MEM non-essential amino acids, 1 mM sodium pyruvate, 1x GlutaMAX, 100 U/mL penicillin and 0.1 mg/mL streptomycin (Sigma or Gibco). Monocytes were stimulated with 100 ng/mL LPS (Sigma), plate-bound immune complex (as previously described [52]), or 100 μg/mL intracellular polyinosinic:polycytidylic acid (poly(I:C)), prepared with 1 mg/mL high molecular weight poly(I:C) mixed 1:1 with LyoVec transfection reagent (Invivogen) at RT for 15 minutes. Where indicated, 1 μg/mL cycloheximide or 5 μg/mL actinomycin D (Sigma) was added to cell cultures. T cells were stimulated with Dynabeads Human T-Activator CD3/CD28 (Life Technologies) at a 1:1 ratio of beads:cells. For the PBMC proliferation assay, five million CD8^-^ cells were stained with Cell Proliferation Dye eFluor 670 (eBioscience), according to the manufacturer’s instructions. These cells were stimulated for 48 hours with 1 μg/mL anti-CD3 (OKT3), with addition of 10 μg/mL blocking anti-TNFSF15 monoclonal antibody (1A9, described under Soluble cytokine measurements) where indicated.

Monocytes were differentiated into monocyte-derived macrophages in the presence of M-CSF or GM-CSF. For M-CSF macrophages, cells were grown in 10 ng/mL recombinant human M-CSF (R and D Systems) for 7 days, adding half the volume of media with 30 ng/mL M-CSF to replenish the cytokine on day 5. For GM-CSF macrophages, monocytes were grown in 50 U/mL recombinant human GM-CSF (Peprotech) for 5 days. Where indicated, macrophages were stimulated with 10 ng/mL LPS for 4 hours. One sample recruited for monocyte-derived macrophage studies was excluded due to poor RNA yield before any measurements were made.

### Nucleic acid extraction and qPCR for samples

RNA and DNA from *ex vivo* and cultured cells was extracted using the AllPrep DNA/RNA Mini Kit (or RNeasy Mini Kit for extracting RNA only), using on-column DNase digestion with the RNase-Free DNase Set (Qiagen). RNA was reverse-transcribed to cDNA using the SuperScript VILO cDNA Synthesis Kit (Life Technologies). qPCR reactions were performed with Taqman Gene Expression Master Mix and Taqman Gene Expression Assays for TNFSF15 (Hs00270802_s1) or Beta-2-Microglobulin (B2M, Hs00984230_m1) (Life Technologies) on an Applied Biosystems 7900HT Fast Real-Time PCR System (Life Technologies) or CFX384 Touch Real-Time PCR Detection System (BioRad). All reactions were performed in triplicate, and the median TNFSF15 Ct value was subtracted from the median B2M Ct value for each sample to generate ΔCt values representing log expression relative to B2M.

### qPCR eQTL analysis

Linear regression test statistics were calculated in R to estimate the effect of each additional copy of the minor (IBD protective) allele on gene expression. To test for an interaction between genotype and stimulation condition, a linear model with coefficients for genotype, stimulation and genotype x stimulation was fitted.

### Genotyping

Samples were genotyped using Taqman SNP Genotyping Assays (rs6478109, C___1305297_10; rs4263839, C____120268_10; rs4246905, C____363307_20; rs7848647, C__11277159_10; rs6478108, C____170492_10) and Taqman Genotyping Master Mix (Life Technologies) according to the manufacturer’s protocol on an Applied Biosystems 7900HT Fast Real-Time PCR System (Life Technologies) or CFX384 Touch Real-Time PCR Detection System (BioRad).

### Allele-specific expression (ASE)

ASE in heterozygous genomic DNA or reverse-transcribed cDNA from the same individual was measured by qPCR. Where ASE was measured in multiple conditions using cells from the same individuals, one genomic DNA sample from each individual was measured concurrently with cDNA samples from multiple conditions. First, the intronic region containing the target SNP was amplified from DNA using the primers and PCR conditions listed in S4 Table. Amplified regions were gel purified. A standard curve was constructed by mixing amplified genomic DNA samples from homozygous individuals 8:1, 4:1, 2:1, 1:1, 1:2, 1:4, and 1:8. Samples were then measured by qPCR in triplicate reactions using Taqman SNP Genotyping Assays for rs4263839 (C____120268_10) or rs4246905 (C____363307_20) and Taqman Gene Expression Master Mix on an Applied Biosystems 7900HT Fast Real-Time PCR System (Life Technologies) or CFX384 Touch Real-Time PCR Detection System (BioRad). Allelic ratios were calculated from VIC and FAM Ct values as described in S1 Text. Unpaired Welch's t-test statistics were calculated using GraphPad Prism Software or R. We used two intronic SNPs, rs4246905 and rs4263839 (the latter of which is in LD r^2^ = 0.977 with rs6478109 and is the most linked of all transcribed SNPs) to examine ASE of *TNFSF15*. Examination of the effect size estimates obtained from assays with each of the two SNP probes on a subset of samples from Fig 2C revealed similar detection of ASE but a slightly lower estimate by the rs4263839 probe (S5B Fig). For this reason, we do not recommend directly comparing effect sizes between samples measured with different probes.

### Soluble cytokine measurements

A mouse monoclonal antibody against human TNFSF15 (clone 1A9) was generated through immunizing mice with human TNFSF15 and screening supernatants for binding TNFSF15 transfected 293T cells. The 1A9 clone also blocks soluble TNFSF15 binding to TNFRSF25 and TNFSF15 costimulation of human T cell activation *ex vivo* [75].

Soluble TNFSF15 in supernatants of stimulated cells was measured by custom Bio-Plex assay. Capture beads were created by conjugating anti-human TNFSF15 antibody (clone 1A9) to Bio-Plex Pro Magnetic COOH beads, region 27, using the Bio-Plex Amine Coupling Kit (BioRad) with 10 μg antibody per reaction, according to the manufacturer’s instructions. Cell culture supernatant assays were performed with 2500 beads per well for capture and 1 μg/mL biotinylated polyclonal rabbit anti-human TNFSF15 (Peprotech) for detection, using the Bio-Plex Pro Reagent Kit (BioRad) and following the manufacturer’s protocol. Data was collected on a Bio-Plex 200 or Bio-Plex MAGPIX Multiplex Reader (BioRad). Cell culture supernatant TNFSF15 concentrations were calculated using a standard curve of recombinant human TNFSF15 (Peprotech).

Serum TNFSF15 levels were also measured by custom Bio-Plex assay. Capture beads were created exactly as described for supernatant cytokine measurements but polyclonal rabbit anti-human TNFSF15 antibody (Peprotech) was used for conjugation to Bio-Plex Pro Magnetic COOH beads, region 27, with 8.5 μg antibody per reaction. Assays were run with samples diluted 1 in 4 in Sample Diluent from the Bio-Plex Pro Reagent Kit (BioRad). Values below the detection limit of the assay were set to 0. Values above the detection limit of the assay were excluded, and this is indicated in the figure legend. For genotype association testing, serum TNFSF15 protein levels were inverse-rank normalized prior to linear regression.

Inflammatory cytokines in the supernatants of stimulated monocytes were measured using the Human ProInflammatory 9-Plex Tissue Culture Kit (MesoScale Diagnostics) at the Core Biochemical Assay Laboratory in Cambridge University Hospitals or using Bio-Plex Pro reagents (BioRad), according to the manufacturer’s protocol. For plotting on a log-scale graph, 2 undetectable IL-10 measurements were set to positive values below the lowest value detected.

Mann-Whitney test statistics were calculated using GraphPad Prism Software.

### Cell surface TNFSF15 measurements

Monocyte surface TNFSF15 expression was measured by flow cytometry. Cells were treated with human FcR Blocking Reagent (Miltenyi) for 5 minutes in PBS and then stained with LIVE/DEAD Fixable Aqua Dead Cell Stain Kit (Invitrogen) and either anti-TNFSF15 (clone 1A9) Alexa-Fluor 647 or mouse IgG2a Alexa-Fluor 647 (BD Biosciences) as an isotype control. Cells were analyzed on a BD LSRFortessa. Mann-Whitney test statistics were calculated using GraphPad Prism Software.

### Ethics statement

The use of genotyped human peripheral blood samples in this study was approved by the National Research Ethics Service, Cambridgeshire 2 Research Ethics Committee (08/H0308/176). All subjects provided written informed consent prior to inclusion in the study.

### Data mining

The data used for corroborating eQTL evidence described in this manuscript were obtained from the GTEx Portal on 10 February 2018 and from the IMMUNPOP browser (http://132.219.138.157/nedelec/eQTL/ and [46]) on 7 March 2018. Regulatory information about the *TNFSF15* promoter region was visualized in the UCSC genome browser (http://genome.ucsc.edu/ and [76], hg19 build) on 17 Feb 2018. Tracks included transcription factor binding sites from ENCODE transcription factor ChIP-seq of 161 factors across a variety of cell types, DNase hypersensitivity signal measured by ENCODE/UW, and CTCF and histone ChIP-seq profiles from ENCODE/BROAD [77]. CAGE data for enhancer identification were downloaded from the ZENBU browser (http://fantom.gsc.riken.jp/zenbu/gLyphs/#config=dXO5cTaJBZiiw73fJq2oGD;loc=hg19::chr9:117566249..117571251+ and [21, 78]), on 17 February 2018.

## Supporting information

S1 Text Supplementary discussion and methods. (PDF)

S1 Fig rs6478109 is a monocyte *TNFSF15* eQTL in healthy controls and inflammatory disease cohorts. (**A**) Plots depict monocyte *TNFSF15* mRNA expression versus rs6478109 genotype in healthy controls and IBD patients (genotyping by Illumina Human OmniExpress12v1.0 BeadChip, gene expression by Affymetrix Human Gene ST 1.1 microarrays). (**B**) Table depicts results of LD calculation and haplotype phasing using 1000 Genomes Phase 3 European (EUR) and East Asian (EAS) cohorts for rs6478109 and rs6478108. Phasing is written as rs6478109 allele 1 in phase with rs6478108 allele 1 / rs6478109 allele 2 in phase with rs6478108 allele 2. (**C**) As (A), plots depict *TNFSF15* mRNA expression versus rs6478108 genotype in healthy controls (n = 39) and IBD patients (n = 80) (genotyping by Illumina Human OmniExpress12v1.0 BeadChip) and patients with ANCA-associated vasculitis (n = 45) (genotyping by Affymetrix SNP 6.0). rs6478109 was not present on the Affymetrix SNP 6.0 genotyping platform, and thus rs6478108 was used as a proxy SNP for examining the eQTL in the AAV cohort. (**D**) *Ex vivo* monocyte *TNFSF15* expression values from Fig 1B plotted by rs6478108 genotype (TT, n = 9; CT, n = 17; CC, n = 9).
(EPS)

S2 Fig The IBD-protective rs6478109:A allele is not associated with monocyte *TNFSF8* expression. Regional association plots for IBD (European ancestry cohort from Liu *et al* [4]) and *TNFSF8* expression (n = 39 healthy individuals and 80 IBD patients). LD is colored with reference to the most associated SNP within each plot.
(EPS)

S3 Fig TNFSF15 expression is induced upon monocyte and T cell stimulation. (**A**) Human peripheral blood monocytes were left unstimulated (null), or were stimulated with 100 ng/mL LPS, cross-linked immune complex, LyoVec transfection reagent control, or LyoVec with 100 μg/mL poly(I:C) for the indicated times. *TNFSF15* mRNA was measured by qPCR relative to *B2M* with expression reported as ΔCt (left); secreted protein was measured in the supernatant by custom Bio-Plex assay (right). Plots are representative of at least 2 independent experiments each. (**B**) As (A) for CD4^+^ and CD8^+^ T cells stimulated with anti-CD3/anti-CD28-coated beads at a ratio of 1:1, beads:cells. The plot on the right shows a longer stimulation time-course for both T cell subsets.
(EPS)

S4 Fig Comparison of eQTL effect sizes in unstimulated and stimulated monocytes. Effect sizes estimated by linear regression (beta coefficients) and their standard errors are plotted for monocyte eQTLs from Fig 1B and Fig 2A.
(EPS)

S5 Fig Genomic regions of interest for allele-specific expression assays. (**A**) The *TNFSF15* gene region was visualized in the UCSC genome browser (hg19 genome build). *TNFSF15* gene position is from RefSeq. SNPs of interest are underlined in the same colors as Fig 4: rs6478109 eQTL SNP in blue, and rs4246905 and rs4263839 SNPs used for allele-specific expression (ASE) measurements in green and orange, respectively. Binding sites for primers used to amplify pre-mRNA for ASE measurements are indicated. (**B**) Probes for rs4246905 and rs4263839 were used to measure ASE in the same four samples of immune complex-stimulated monocytes (samples included in Fig 2C). 95% confidence interval for difference in mean log_2_(allelic ratio) between genomic DNA and cDNA calculated from Welch’s t-test.
(EPS)

S6 Fig rs6478109 genotype is not associated with TNFSF15 protein expression in serum or monocyte expression of other inflammatory cytokines. (**A**) Serum TNFSF15 was measured in rs6478109 genotyped individuals (GG, n = 22; GA, n = 26; AA, n = 19; two additional AA individuals were excluded due to measurements above the detectable range of the standard curve). Line denotes the median; p value from linear regression on inverse-rank normalized values. (**B**) Inflammatory cytokines were measured in supernatants of stimulated monocytes from rs6478109 homozygotes (immune complex, n = 4 per genotype; LPS, n = 7 per genotype). No significant differences between genotypes by Mann-Whitney test. (**C**) Selected cytokines from (B) were further examined in a second cohort (n = 12 per genotype). No significant differences between genotypes by Mann-Whitney test.
(EPS)

S7 Fig Chromatin modifications and transcription factor binding at the *TNFSF15* promoter. The promoter region of *TNFSF15* was visualized in the UCSC genome browser (http://genome.ucsc.edu/ and [76], hg19 build). *TNFSF15* gene position is from RefSeq. SNPs of interest are indicated. Genome regulatory marks from ENCODE [77] are shown. Transcription factor binding sites are from ENCODE transcription factor ChIP-seq of 161 factors across a variety of cell types with consensus motifs marked in green. Histogram tracks represent measurements in primary human monocytes. “DHS” denotes raw DNase hypersensitivity signal measured by ENCODE/UW. CTCF and histone ChIP-seq profiles highlighted in orange are from ENCODE/BROAD. “Control” indicates sequencing of ChIP input control DNA. Enhancers and transcriptional activity identified by Cap Analysis of Gene Expression (CAGE) were downloaded from the ZENBU browser view associated with Baillie *et al* [21] (http://fantom.gsc.riken.jp/zenbu/gLyphs/#config=dXO5cTaJBZiiw73fJq2oGD;loc=hg19::chr9:117566249..117571251+), which includes FANTOM5 consortium data [78]. CAGE-identified enhancers across multiple human cell types and monocyte-derived macrophages, as well as transcriptional activity measurements by CAGE, are highlighted in turquoise.
(EPS)

S8 Fig Immunophenotyping gating strategies. Immunophenotyping gating strategies are depicted for (**A**) basic T cell subsets, (**B**) Th effector cell subsets, (**C**) Treg, (**D**) B cells, and (**E**) monocytes, dendritic cells, and NK cells.
(EPS)

S9 Fig Immunophenotypes are not associated with rs6478109 genotype. Age- and sex-matched rs6478109 homozygotes were immunophenotyped for 39 different peripheral blood parameters. Between 6 and 19 pairs were examined for each subset. No immune cell subsets were found to be differentially abundant between genotypes by Wilcoxon test.
(EPS)

S10 Fig rs6478109-*TNFSF15* eQTL in MDM from Nedelec *et al*. eQTLs in M-CSF-differentiated MDM with and without infection [46]. Plots were generated using the IMMUNPOP browser at http://132.219.138.157/nedelec/eQTL/
(EPS)

S11 Fig LD at the *TNFSF15* locus. Regional linkage disequilibrium (r^2^) plot of genetic variants +/- 3 kbp from rs6478109 calculated using 1000 Genomes Phase 3 EUR cohort.
(EPS)

S1 Table rs6478109-*TNFSF15* eQTL statistics from measurements on resting and stimulated cells from bioresource volunteers. (PDF)

S2 Table Allele-specific expression statistics from measurements on stimulated cells from rs6478109 heterozygous bioresource volunteers. (PDF)

S3 Table Evaluation of potential candidate SNPs at the *TNFSF15* IBD susceptibility locus. (PDF)

S4 Table Primers used for pre-amplification in allele-specific expression assays. (PDF)

S5 Table Antibody panels for immunophenotyping. (PDF)

S1 Data Numeric data from eQTL, ASE, protein expression and T cell activation measurements underlying Figs 1B, 2, 3A, 3B, 4, 5 and 6. (ZIP)
