# Supplementary material for: Reduced monocyte and macrophage TNFSF15/TL1A expression is associated with susceptibility to inflammatory bowel disease
Source: PLoS Genet. 2018 Sep 10;14(9):e1007458. doi: 10.1371/journal.pgen.1007458 (PMC6130856; doi:10.1371/journal.pgen.1007458)
Supplement: S1 Text — (PDF) [file pgen.1007458.s001.pdf]

S1 Text

Discussion of cell-type specificity of the eQTL at *TNFSF15*

While we cannot formally exclude the possibility of a *TNFSF15* eQTL with a small effect size that we were under-powered to detect in stimulated T cells, we do not have evidence to reject the null hypothesis of no eQTL. In our recall-by-genotype eQTL analysis (Fig 2B), we had 10 versus 9 samples homozygous for each allele in stimulated $CD4^+$ T cells and 9 versus 9 in stimulated $CD8^+$ T cells. We conducted a t-test power calculation to determine the effect size (here, change in mean expression between minor and major allele homozygotes divided by the pooled standard deviation) that we would be able to detect at a significance threshold of p 0.05. We emphasize that the effect size described here is distinct from the beta estimate from eQTL linear regression. We determined that we had 80% power to detect an effect size of 1.37 and 1.41 in stimulated CD4+ T cells and CD8+ T cells, respectively. For comparison, the observed effect size (as defined above) in *ex vivo* monocytes, where we did find a significant eQTL, was 2.58. In addition to conventional eQTL analysis, we also examined allele-specific expression (ASE) in stimulated T cells from heterozygous individuals. This assay compares the ratio of transcripts within each heterozygous individual and is therefore not affected by inter-individual variation in gene expression, thus providing greater power. Even with the ASE assay, we found no significant allelic imbalance in either stimulated T cell subset. Furthermore, visual inspection of the ASE plots did not support an eQTL. Therefore, our data point to a strong monocyte eQTL but do not support a substantial stimulated T cell eQTL.

Additional discussion of previous functional studies of the IBD risk locus at *TNFSF15*

Previous studies describing gene expression association with the IBD-associated locus at *TNFSF15* have yielded conflicting results. We identified an association of decreased monocyte TNFSF15 expression with the IBD risk allele. We here discuss how our data relate to additional previous studies not described in the main text.

Our eQTL results are concordant with the data from two previous reports by Michelsen *et al* [1] and Sun *et al* [2], but these studies incorrectly attributed IBD risk to the opposite allele. Michelsen *et al* [1] focused on the Ashkenazi Jewish population where CD susceptibility

haplotypes at *TNFSF15* were suggested to be reversed [3], but subsequent genetic association studies in this population have not found such an association [4, 5]. Thus, the haplotype associated with increased TNFSF15 expression in Michelsen *et al* is the haplotype associated with IBD protection in the general European population, which we also find to be associated with elevated TNFSF15. Sun *et al* [2] examined genotype association with mRNA from whole blood in a healthy Chinese cohort and reported the rs6478109:A allele to be associated with increased *TNFSF15* expression, consistent with our results. However, they reported this allele to be part of the IBD risk haplotype; this risk attribution is inconsistent with previous GWAS results where rs6478109:A has been directly reported as protective for CD in Japanese individuals [6], as well as in the Liu *et al* GWAS meta-analysis and a previous studiy of pediatric onset IBD in European individuals [7, 8]. Furthermore, the T risk allele that Sun *et al* reported at rs6478108, the SNP most strongly associated with CD in their study [2], is out of phase with the A risk allele reported at the eQTL SNP rs6478109 in both East Asian and European individuals (S1B Fig), suggesting an error in their analysis. Together, these results clearly indicate that the protective allele rs64718109:A is associated with increased monocyte TNFSF15 expression as we have demonstrated.

Two additional small studies have reported the opposite direction of effect of the *TNFSF15* eQTL. Zucchelli *et al* observed increased *TNFSF15* mRNA in peripheral blood leukocytes (n=25) and rectal biopsies (n=15) from healthy donors with the rs4263839:G risk allele (in phase with the rs6478109-G risk allele) [9]. However, it is difficult to compare these data from mixed cell populations with our data from purified monocytes, and findings from a small sample size must be interpreted with caution. In contrast to a report by Kakuta *et al*, we found no allele-specific expression of *TNFSF15* in stimulated T cells [10]. Kakuta *et al* activated whole PBMC with PHA and total T cells with PMA and ionomycin [10], whereas we activated separate populations of $CD4^+$ and $CD8^+$ T cells with anti-CD3 and anti-CD28. It is possible that the results differ due to cell type composition or stimulus, but we believe that our additional purification provides strong evidence against a substantial T cell eQTL.

Finally, two large-scale studies found a *TNFSF15* eQTL at the same narrowed LD block that we identified in resting monocytes [11] and influenza-stimulated monocyte-derived dendritic cells [12], but the reference allele was not specified in either study.

Supplementary Methods

Assays with elutriated monocytes and cells sorted from leukocyte cones:

Genotype-independent assays were performed using positively selected subsets from leukocyte cones obtained through the National Health Service (NHS) Blood and Transplant service at Addenbrooke's Hospital or elutriated monocytes from the National Institutes of Health Department of Transfusion Medicine. Leukocyte cones were processed as described in Methods "Whole blood processing". Elutriated monocytes were passed through a 100 µm strainer and washed in PBS before plating.

Linkage disequilibrium (LD) calculation:

LD between SNPs was calculated from the 1000 Genomes phase 3 European (EUR) cohort samples and the 1000 Genomes phase 3 East Asian (EAS) cohort samples [13]. VCF files were converted to PLINK format using vcftools [14], and LD calculated for desired SNPs in PLINK v1.9 [15, 16]. LD matrix for S11 Fig calculated by https://analysistools.nci.nih.gov/LDlink/?tab=ldmatrix and plotted using the "gaston" R package.

Allele-specific expression allelic ratio calculation:

Allelic ratios were calculated from VIC and FAM Ct values by fitting a line to the standard curve according to the following rationale. For allele F amplified by the FAM-labeled probe with efficiency E_F from initial concentration F_i to cross the threshold concentration at cycle Ct_F, and likewise for allele V amplified by the VIC-labeled probe,

$$F_i * E_F^{Ct_F} = threshold = V_i * E_V^{Ct_V}$$

Rearranging and taking the natural logarithm reveals that

$$\ln\left(\frac{F_i}{V_i}\right) = Ct_V \ln\left(E_V\right) - Ct_F \ln\left(E_F\right)$$

Assuming equal efficiencies for VIC and FAM probe amplification increases robustness to pipetting error as each VIC versus FAM comparison may be calculated within each individual reaction. Thus with this assumption

$$\ln\left(\frac{F_i}{V_i}\right) = \left(Ct_V - Ct_F\right) * \ln\left(E\right)$$

We fitted a line to this equation using the Ct values from VIC and FAM probes in the standard curve dilutions. This intercept and slope were then used to then calculate starting allelic ratios in the cDNA and gDNA samples. To adjust for slight measurement errors possible with such dilute DNA samples in the standard curve, all allele-specific expression ratios were normalized to the average gDNA ratio measured in the individual assay.

Immunophenotyping:

Flow cytometry immunophenotyping of PBMC was performed using 5 different panels, as detailed in S5 Table and S8 Fig. Homozygous major and homozygous minor individuals were recruited on the same day and sex-matched and age-matched within 5 years to control for immunological parameters that change with these traits. Two individuals were found to not down-regulate CD45RA and were thus excluded with their homozygous pairs from categories relying on this surface antigen. Flow cytometry was performed on a BD LSR Fortessa (BD Biosciences) and data analyzed in FlowJo (FlowJo, LLC). Wilcoxon test statistics were calculated using GraphPad Prism Software.

Power calculation:

Power calculations for two samples t-tests using samples of different sizes were performed using the pwr.t2n.test function of the pwr R package.

Supplementary Material References:

1. Michelsen KS, Thomas LS, Taylor KD, Yu QT, Mei L, Landers CJ, et al. IBD-associated TL1A gene (TNFSF15) haplotypes determine increased expression of TL1A protein. PLoS One. 2009;4(3):e4719.
2. Sun Y, Irwanto A, Toyo-Oka L, Hong M, Liu H, Andiappan AK, et al. Fine-mapping analysis revealed complex pleiotropic effect and tissue-specific regulatory mechanism of TNFSF15 in primary biliary cholangitis, Crohn's disease and leprosy. Sci Rep. 2016;6:31429.
3. Picornell Y, Mei L, Taylor K, Yang H, Targan SR, Rotter JI. TNFSF15 is an ethnic-specific IBD gene. Inflamm Bowel Dis. 2007;13(11):1333-8.
4. Kenny EE, Pe'er I, Karban A, Ozelius L, Mitchell AA, Ng SM, et al. A genome-wide scan of Ashkenazi Jewish Crohn's disease suggests novel susceptibility loci. PLoS Genet. 2012;8(3):e1002559.
5. Peter I, Mitchell AA, Ozelius L, Erazo M, Hu J, Doheny D, et al. Evaluation of 22 genetic variants with Crohn's disease risk in the Ashkenazi Jewish population: a case-control study. BMC Med Genet. 2011;12:63.
6. Yamazaki K, McGovern D, Ragoussis J, Paolucci M, Butler H, Jewell D, et al. Single nucleotide polymorphisms in TNFSF15 confer susceptibility to Crohn's disease. Hum Mol Genet. 2005;14(22):3499-506.
7. Kugathasan S, Baldassano RN, Bradfield JP, Sleiman PM, Imielinski M, Guthery SL, et al. Loci on 20q13 and 21q22 are associated with pediatric-onset inflammatory bowel disease. Nat Genet. 2008;40(10):1211-5.
8. Liu JZ, van Sommeren S, Huang H, Ng SC, Alberts R, Takahashi A, et al. Association analyses identify 38 susceptibility loci for inflammatory bowel disease and highlight shared genetic risk across populations. Nat Genet. 2015;47(9):979-86.
9. Zucchelli M, Camilleri M, Andreasson AN, Bresso F, Dlugosz A, Halfvarson J, et al. Association of TNFSF15 polymorphism with irritable bowel syndrome. Gut. 2011;60(12):1671-7.
10. Kakuta Y, Ueki N, Kinouchi Y, Negoro K, Endo K, Nomura E, et al. TNFSF15 transcripts from risk haplotype for Crohn's disease are overexpressed in stimulated T cells. Hum Mol Genet. 2009;18(6):1089-98.
11. Raj T, Rothamel K, Mostafavi S, Ye C, Lee MN, Replogle JM, et al. Polarization of the effects of autoimmune and neurodegenerative risk alleles in leukocytes. Science. 2014;344(6183):519-23.
12. Lee MN, Ye C, Villani AC, Raj T, Li W, Eisenhaure TM, et al. Common genetic variants modulate pathogen-sensing responses in human dendritic cells. Science. 2014;343(6175):1246980.
13. Auton A, Brooks LD, Durbin RM, Garrison EP, Kang HM, Korbel JO, et al. A global reference for human genetic variation. Nature. 2015;526(7571):68-74.
14. Danecek P, Auton A, Abecasis G, Albers CA, Banks E, DePristo MA, et al. The variant call format and VCFtools. Bioinformatics. 2011;27(15):2156-8.
15. Chang CC, Chow CC, Tellier LC, Vattikuti S, Purcell SM, Lee JJ. Second-generation PLINK: rising to the challenge of larger and richer datasets. Gigascience. 2015;4:7.
16. Purcell S, Chang C. PLINK [version 1.9] . www.cog-genomics.org/plink/1.9/.
