# Supplementary material for: Reduced monocyte and macrophage TNFSF15/TL1A expression is associated with susceptibility to inflammatory bowel disease
Source: PLoS Genet. 2018 Sep 10;14(9):e1007458. doi: 10.1371/journal.pgen.1007458 (PMC6130856; doi:10.1371/journal.pgen.1007458)
Supplement: S1 Table — (PDF) [file pgen.1007458.s013.pdf]

S1 Table:

rs6478109-*TNFSF15* eQTL statistics from measurements on resting and stimulated cells from bioresource volunteers

	n (GG)	n (GA)	n (AA)	beta	SE	95% CI	p-value
**eQTL genotype term**							
monocytes - *ex vivo*	10	16	9	0.707	0.131	(0.441, 0.974)	5.63E-06
monocytes - immune complex stimulation	6	10	5	0.923	0.246	(0.409, 1.437)	1.33E-03
monocytes - intracellular poly(I:C) stimulation	8	10	8	0.368	0.141	(0.076, 0.659)	1.55E-02
monocytes - LPS stimulation	8	11	7	0.478	0.426	(-0.401, 1.358)	2.73E-01
CD4+ T cells - anti-CD3/anti-CD28 stimulation	10	16	9	-0.026	0.149	(-0.329, 0.276)	8.61E-01
CD8+ T cells - anti-CD3/anti-CD28 stimulation	9	10	9	-0.098	0.199	(-0.507, 0.311)	6.27E-01

**eQTL genotype x stimulation interaction term**					
monocytes - immune complex stimulation *v ex vivo*	n combined from relevant categories above	0.216	0.254	(-0.294, 0.725)	3.99E-01
monocytes - intracellular poly(I:C) stimulation *v ex vivo*		-0.229	0.399	(-1.028, 0.570)	5.68E-01
monocytes - LPS stimulation *v ex vivo*		-0.340	0.193	(-0.726, 0.046)	8.33E-02

This table provides eQTL statistics from linear regression of the data shown in Fig 2A,B. In the top half of the table, *TNFSF15 v B2M* ΔCt values were regressed on minor allele dosage using simple linear regression. Linear regression analyses were performed separately for each cell type and condition. Beta estimates are reported for the genotype term. In the bottom half of the table, for each stimulation condition, data from unstimulated and stimulated monocytes were considered together, regressing *TNFSF15 v B2M* ΔCt values on minor allele dosage, stimulation condition, and the interaction of allelic dosage and stimulation. In these analyses, all genotype and stimulation terms were significant. Beta estimates are reported for the interaction term of each comparison.
