# Supplementary material for: Reduced monocyte and macrophage TNFSF15/TL1A expression is associated with susceptibility to inflammatory bowel disease
Source: PLoS Genet. 2018 Sep 10;14(9):e1007458. doi: 10.1371/journal.pgen.1007458 (PMC6130856; doi:10.1371/journal.pgen.1007458)
Supplement: S2 Table — (PDF) [file pgen.1007458.s014.pdf]

S2 Table:

Allele-specific expression statistics from measurements on stimulated cells from rs6478109 heterozygous bioresource volunteers depicted in Fig 2 C-D.

	n	ASE SNP	μ cDNA – μ gDNA	95% CI	p-value
monocytes - immune complex stimulation	7	rs4246905	1.282	(1.083, 1.482)	1.09E-06
monocytes - intracellular poly(I:C) stimulation	10	rs4263839	0.434	(0.079, 0.789)	2.15E-02
monocytes - LPS stimulation	12	rs4246905	1.039	(0.721, 1.358)	1.46E-05
CD4+ T cells - anti-CD3/anti-CD28 stimulation	11	rs4246905	-0.177	(-0.447, 0.092)	1.75E-01
CD8+ T cells - anti-CD3/anti-CD28 stimulation	10	rs4263839	-0.093	(-0.340, 0.154)	4.24E-01
