# Supplementary material for: Reduced monocyte and macrophage TNFSF15/TL1A expression is associated with susceptibility to inflammatory bowel disease
Source: PLoS Genet. 2018 Sep 10;14(9):e1007458. doi: 10.1371/journal.pgen.1007458 (PMC6130856; doi:10.1371/journal.pgen.1007458)
Supplement: S3 Table — (PDF) [file pgen.1007458.s015.pdf]

S3 Table:
Evaluation of potential candidate SNPs at the *TNFSF15* IBD susceptibility locus

**SNP[A,B]**	**Location**	**LD r^2 with rs4246905**	**LD r^2 with rs4263839**	**LD r^2 with rs6478109**
rs6478108	intron 1	0.599	0.939	0.917
rs4372078	intron 1	0.722	0.873	0.854
rs4366152	intron 1	0.634	1.000	0.977
rs4263839	intron 1	0.634	1.000	0.977
rs6478109	*upstream*	*0.618*	*0.977*	*1.000*
rs7848647	*upstream*	*0.615*	*0.973*	*0.995*
rs35396782	upstream	0.668	0.828	0.817
rs10817678	*upstream*	*0.580*	*0.925*	*0.947*
rs7869487	upstream	0.631	0.772	0.761
rs722126	upstream	0.506	0.759	0.749
rs1853187	upstream	0.391	0.699	0.695
rs4979464	upstream	0.391	0.699	0.695
rs7040029	upstream	0.495	0.733	0.723
rs1467563	upstream	0.472	0.740	0.730
rs6478113	upstream	0.355	0.640	0.659
rs7866342	upstream	0.472	0.740	0.730
rs911605	upstream	0.416	0.640	0.630

Table lists SNPs that are both in greater LD with rs6478109 and rs4263839 than with rs4246905 and are in greater LD with rs6478109 and rs4263839 than is rs4246905. LD calculations were made using the 1000 Genomes Phase 3 EUR cohort.

[A] Colored SNPs correspond to SNPs in Fig 4.

[B] Italicized, underlined SNPs are those in high LD with rs6478109 (r^2 > 0.8) that are in greater LD with rs6478109 than with rs4263839.
