# Supplementary material for: Reduced monocyte and macrophage TNFSF15/TL1A expression is associated with susceptibility to inflammatory bowel disease
Source: PLoS Genet. 2018 Sep 10;14(9):e1007458. doi: 10.1371/journal.pgen.1007458 (PMC6130856; doi:10.1371/journal.pgen.1007458)
Supplement: S4 Table — (PDF) [file pgen.1007458.s016.pdf]

S4 Table:

Primers used for pre-amplification in allele-specific expression assay

SNP	Primer names	Sequences (5’-3’)	Fragment Size
rs4246905	TL1A-rs4246905-F<br>TL1A-rs4246905-R	TGCTGTGTGGGAGTTTGTCT<br>TCATTCCTCCCCAAAGCAGT	164 bp
rs4263839	TL1A-rs4263839-F<br>TL1A-rs4263839-R	ACCCCCATTTCCCTCTCCTT<br>ACTCACTGCTACTGCCCCTA	208 bp
