# Supplementary material for: Reduced monocyte and macrophage TNFSF15/TL1A expression is associated with susceptibility to inflammatory bowel disease
Source: PLoS Genet. 2018 Sep 10;14(9):e1007458. doi: 10.1371/journal.pgen.1007458 (PMC6130856; doi:10.1371/journal.pgen.1007458)
Supplement: S5 Table — (PDF) [file pgen.1007458.s017.pdf]

S5 Table:

Antibody panels for human immunophenotyping

Panel	Target	Clone	Color	Company
T cells – basic	Live/Dead		Blue	Life Technologies
	CD3	SK7	Am-Cyan	BD Biosciences
	CD4	RPA-T4	APC-eFluor 780	eBioscience
	CD8	RPA-T8	Pacific Blue	BD Biosciences
	CD45RA	HI100	PerCP-Cy5.5	eBioscience
	CD27	O323	PE-Cy7	eBioscience
	CCR7	150503	PE	BD Biosciences
T cells – Th subsets	Live/Dead		Aqua	Life Technologies
	CD3	OKT3	eFluor 650-NC	eBioscience
	CD4	RPA-T4	APC-eFluor 780	eBioscience
	CD8	RPA-T8 or SK1	Pacific Blue or eFluor 450	BD Biosciences or eBioscience
	CD45RA	HI100	PerCP-Cy5.5	eBioscience
	CCR7	150503	PE	BD Biosciences
	CXCR3	49801	FITC	R&D Systems
	CCR6	R6H1	PE-Cy7	eBioscience
T cells – Treg	Live/Dead		Aqua	Life Technologies
	CD3	OKT3	eFluor 650-NC	eBioscience
	CD4	RPA-T4	APC-eFluor 780	eBioscience
	CD8	RPA-T8 or SK1	Pacific Blue or eFluor 450	BD Biosciences or eBioscience
	CD45RA	HI100	PerCP-Cy5.5	eBioscience
	CCR4	1G1	PE-Cy7	BD Biosciences

	CD25	M-A251	PE	BD Biosciences
	CD127	hIL-7R-M21	Alexa-Fluor 647	BD Biosciences
B cells	Live/Dead		Blue	Life Technologies
	CD20	2H7	APC-eFluor 780	eBioscience
	CD19	HIB19	V450	BD Biosciences
	CD38	HB7	eFluor 650-NC	eBioscience
	CD24	ML5	PerCP-Cy5.5	BD Biosciences
	CD27	O323	PE-Cy7	eBioscience
	IgD	IA6-2	FITC	BD Biosciences
monocytes, dendritic cells, NK cells	Live/Dead		Blue or Aqua	Life Technologies
	CD20	2H7	APC-eFluor 780	eBioscience
	CD19	HIB19	APC-eFluor 780	eBioscience
	CD3	SK7 or OKT3	Am-Cyan or eFluor 650-NC	BD Biosciences or eBioscience
	CD11c	3.9	PE-Cy7	eBioscience
	CD123	6H6	PerCP-Cy5.5	eBioscience
	CD14	61D3	eFluor 605-NC	eBioscience
	CD16	3G8 or eBioCB16	Pacific Blue or APC	BD Biosciences or eBioscience
	CD56	MEM-188	FITC	eBioscience
	HLA-DR	G46-6 or L243	PE or eFluor 450	BD Biosciences or eBioscience
